# Structural insight into LexA–RecA* interaction

**DOI:** 10.1093/nar/gkt744

**Published:** 2013-08-21

**Authors:** Lidija Kovačič, Nejc Paulič, Adrijana Leonardi, Vesna Hodnik, Gregor Anderluh, Zdravko Podlesek, Darja Žgur-Bertok, Igor Križaj, Matej Butala

**Affiliations:** ^1^Department of Molecular and Biomedical Sciences, Jožef Stefan Institute, Jamova 39, 1000 Ljubljana, Slovenia, ^2^Department of Biology, University of Ljubljana, Biotechnical Faculty, Večna pot 111, 1000 Ljubljana, Slovenia, ^3^National Institute of Chemistry, 1000 Ljubljana, Slovenia, ^4^Department of Chemistry and Biochemistry, Faculty of Chemistry and Chemical Technology, University of Ljubljana, Aškerčeva 5, SI-1000 Ljubljana, Slovenia and ^5^Centre of Excellence for Integrated Approaches in Chemistry and Biology of Proteins, Jamova 39, 1000 Ljubljana, Slovenia

## Abstract

RecA protein is a hallmark for the bacterial response to insults inflicted on DNA. It catalyzes the strand exchange step of homologous recombination and stimulates self-inactivation of the LexA transcriptional repressor. Importantly, by these activities, RecA contributes to the antibiotic resistance of bacteria. An original way to decrease the acquisition of antibiotic resistance would be to block RecA association with LexA. To engineer inhibitors of LexA–RecA complex formation, we have mapped the interaction area between LexA and active RecA–ssDNA filament (RecA*) and generated a three-dimensional model of the complex. The model revealed that one subunit of the LexA dimer wedges into a deep helical groove of RecA*, forming multiple interaction sites along seven consecutive RecA protomers. Based on the model, we predicted that LexA in its DNA-binding conformation also forms a complex with RecA* and that the operator DNA sterically precludes interaction with RecA*, which guides the induction of SOS gene expression. Moreover, the model shows that besides the catalytic C-terminal domain of LexA, its N-terminal DNA-binding domain also interacts with RecA*. Because all the model-based predictions have been confirmed experimentally, the presented model offers a validated insight into the critical step of the bacterial DNA damage response.

## INTRODUCTION

Factors that cause DNA damage or block replication forks induce a number of co-ordinately regulated responses in bacteria, including the SOS response (1). The latter encompasses an inducible DNA damage response with functions for maintaining genetic integrity and enhancing adaptation of bacteria through either increased mutation rate or accelerated genome re-arrangements, with the consequent spread of mobile genetic elements (2–4). Because sub-lethal doses of several therapeutically used antibiotics induce DNA damage, antibiotic therapy can be counteracted at many levels by the bacterial SOS response (5). Hence, selective inhibitors of SOS induction can enhance the effectiveness of antibiotic chemotherapy (3).

The SOS response in *Escherichia coli* is controlled by LexA, a global transcriptional repressor, and RecA, an inducer of transcription (6). Functional LexA is a homodimer that interacts with specific DNA sequences (operator DNA) via a helix-turn-helix motif in its amino-terminal domain (NTD). LexA is a latent serine proteinase. Its dimerization and self-cleaving activity determinants are located within its carboxy-terminal domain (CTD) (7). RecA is activated (RecA*) by binding Mg^2+^ ions and ATP, and polymerizing on a single-stranded DNA (ssDNA) formed at sites of the DNA damage (8,9). The repressor is stable in normally growing cells, but its intramolecular self-cleaving activity is triggered by interaction with RecA* (10). LexA can adopt a cleavable conformation, in which the specific cleavage site, the Ala84–Gly85 bond, is positioned close to the catalytic dyad Ser119 and Lys156, or a non-cleavable conformation with this bond about 20 Å distant (7). It was suggested that RecA* stabilizes LexA in the cleavable state (7), but recent results imply that it induces a conformational change of the cleavage site region in the repressor (11). Despite several mutational studies and electron microscopic analysis, the specific residues that compose the LexA–RecA* interface remain elusive (12,13). The LexA binding site presumably extends from the RecA* Walker A motif, which co-ordinates the ATP phosphate groups and the Mg^2+^ ion, along the L1 loop region (residues 157–165) that binds ssDNA to the region in which residue Arg243 is positioned (8,14). It was proposed recently that repressor CTDs bind across the filament groove by interacting with the RecA residues Gly22 and Gly108, while its DNA-binding NTDs interact with the RecA* core region (15).

Self-cleavage commences only after LexA has dissociated from operator DNA, and leads to complete degradation of the C- and N-terminal LexA fragments (10), as repressor bound to operator DNA cannot interact with RecA* (16). Thus, diversity within LexA operators instigates the timing of induction of SOS genes, leading to co-ordinated de-repression of >30 promoters in *E. coli* (17). Preventing the association between LexA and RecA* by specific inhibitors would block the bacterial SOS response during antibiotic therapy and limit the acquisition of antibiotic resistance (3,18,19). Precise definition of the interaction between LexA and RecA* could therefore enable the design of drugs to combat the antibiotic resistance of bacteria.

To define the binding region between the key interacting SOS players, we cross-linked the active nucleoprotein filament RecA* to the repressor LexA, and identified the cross-linked parts of the two proteins. Mapping of the binding region between RecA* and LexA led to generation of a three-dimensional model of the complex. The results indicate that one subunit of the LexA dimer interacts with RecA protomers at positions *i* to *i* + 7 along a deep helical groove of the filament. The second subunit of the repressor has only minimal contacts with RecA*. Unexpectedly, the model also permitted complex formation with the repressor in an operator-binding conformation, however, without DNA attached. By engineering a LexA mutant that adopts the operator-binding state and by experimental confirmation of the formation of the complex with RecA*, the RecA*–LexA three-dimensional model was validated. Also predicted by the model, binding of the operator DNA to LexA sterically hinders association of the latter with the active nucleoprotein filament. In this way, the mechanism that modulates the de-repression of the SOS genes has been defined.

## MATERIALS AND METHODS

### Proteins

Proteins were purified as described (16). The pJWL904 plasmid for overexpressing the His-tagged LexA protein harbouring mutations L89P, Q92W, E152A and K156A, the quadruple LexA mutant (LexAQM) and the plasmid encoding the LexA(K156A) mutant were gifts from Dr J.W. Little. The mutation K156A renders the repressor non-cleavable while modification of the other three residues in LexAQM promotes RecA*-independent cleavage (7). Three DNA fragments encoding LexA mutants harbouring 4, 5 and 9 amino acid substitutions, respectively (Supplementary Table S1), and the flanking BamHI–MluI sites were provided by GeneScript (USA). These DNA fragments were used to prepare plasmids pLexA4, pLexA5, pLexA9 overexpressing LexA4, LexA5 or LexA9 protein. Amino acid residues were selected for substitution based on the energetically most favourable generated models on the criterion that they lie at the LexA-RecA* interface and increase the stability of the complex, and that they had been shown not to affect the LexA cleavage reaction (6). The High Ambiguity Driven protein–protein Docking (HADDOCK) approach was used to test the effect of mutations on the *in silico* stability of the complex (20). Circular dichroism (CD) spectroscopy was used to indicate changes in secondary and/or tertiary structure of isolated repressor variants from that of the wild-type (wt) LexA (all in 8 µM concentration), measured on an Aviv 75 spectropolarimeter, using parameters as described earlier (21). Mixture of primers LexA_u (16) and 69D (5′-cgcacgcgtttacaacagacgaatcccgcgtgat-3′) was used to PCR amplify the BamHI–MluI flanking fragments and to prepare the pLexA69 overexpression plasmid harbouring the *lexA* sequence coding for LexA residues 1–69. The plasmid variant of the LexA repressor construct plasmid pAna1 (16) to overexpress the LexA(M24C) protein, pLexAM24C, was engineered based on the DNA-bound LexA structure (22) and constructed according to the QuickChange® Site-directed Mutagenesis Kit manual (Stratagene) using primers M24CUP (5′-atcacatcagccagacaggttgcccgccgacgcgt-3′) and M24CDO (5′-acgcgtcggcgggcaacctgtctggctgatgtgat-3′). All the proteins were >95% pure following Ni–NTA affinity chromatography. The LexA(M24C) mutant was treated with 20 mM dithiothreitol (DTT) or with 0.5 mM 1,10-phenanthroline and 0.1 mM CuSO_4_ for 30 min at room temperature to obtain reduced or oxidized mutants, respectively. PD10 desalting columns (GE Healthcare) were used for exchange of the buffer. The proteins were stored at −80°C in 20 mM NaH_2_PO_4_ (pH 7.3), 0.2 M NaCl. Concentrations of proteins were determined using NanoDrop1000 (Thermo SCIENTIFIC) and extinction coefficients at 280 nm of 6990 M^−^^1 ^cm^−^^1^ for LexA and LexA(M24C), 12 490 M^−^^1 ^cm^−^^1^ for LexAQM and 21 555 M^−^^1 ^cm^−^^1^ for RecA. Concentration of LexA(1–69) protein was measured using the BCA protein assay kit (Pierce).

### Repressor cleavage assay

Self-cleavage of the wt repressor and its LexA4, LexA5 variants was induced at alkaline pH in 100 mM 3-(cyclohexylamino)-1-propanesulphonic acid-NaOH (CAPS) (pH 9.5), 200 mM NaCl at 37°C. RecA*-induced self-cleavage of wt LexA, LexA4, LexA5 and of reduced or oxidized LexA(M24C) (all at 2 µM concentration) was performed at 37°C as described (11). Oxidized or reduced LexA(M24C) was preincubated with either non-specific or specific DNA (∼1.5 µM) for 5 min at 37°C before induction of the RecA*-mediated self-cleavage. To prepare non-specific DNA or a DNA fragment with *tisB* operator, complementary primers NonUp (5′-tttaccacataaataagtggtaat-3′) and NonDo (5′-attaccacttatttatgtggtaaa-3′) or TisBUp (5′-attactgtttatttatacagtaaa-3′) and TisBDo (5′-tttactgtataaataaacagtaat-3′), respectively, in 20 mM Tris–HCl (pH 7.3), 0.14 M NaCl were mixed in a 1:1 (mol:mol) ratio. Primers were annealed in a temperature gradient from 94°C to 4°C (∼1.5 h) in a PCR machine (Eppendorf). For the LexA(M24C) cleavage reactions, the LexA(M24C) dimer to operator/modified operator ratio was 1:2. Oxidized or reduced protein was preincubated with specific or non-specific DNA for 5 min at 37°C after which RecA* was added to initiate the reaction. Cleavage reactions were performed in 20 mM Tris (7.4), 40 mM NaCl, 5 mM MgCl_2_, 1 mM ATP-γ-S, 1 mM DTT, 2% (w/v) glycerol. 4×NuPAGE LDS sample buffer (Invitrogen) was used to stop the reactions. The products were analysed on 12% NuPAGE gels in MES running buffer (Invitrogen) and stained by PAGE blue protein stain (Fermentas). The experiments were conducted three times, and a representative gel is shown. The bands stemming from LexA(M24C) were quantified using a G:Box (Syngene). The integrated optical densities of the oxidized LexA(M24C) dimer or the reduced LexA(M24C) monomer were determined as a function of time. Changes in protein levels are presented as the ratio of the optical density at the time zero relative to that of the sample at times 5 and 15 min after the addition of RecA* to the reaction mixture.

### Preparation of sulpho-SBED–LexA–RecA* conjugate

Sulpho**-**SBED is a commercially available hetero-, bifunctional cross**-**linker that contains a sulphonated N**-**hydroxysuccinimide amino**-**reactive group and a photoactivatable aryl azide functionality that forms, on photolysis, a short-lived nitrene that reacts with nucleophiles, especially amines. Sulpho**-**SBED also contains a biotin moiety, which allows specific avidin/streptavidin**-**based purification or detection of labelled proteins and peptides, and a cleavable disulphide bond in the spacer between the two reactive groups. This allows label transfer between proteins in a complex (Supplementary Figure S1A).

RecA protein (42 µM) was incubated on ice for 2 h with single-stranded DNA (9.2 µM) (SKBT25, 5′-gcgtgtgtggtggtgtgc-3′) (11), a nucleotide cofactor ATP-γ-S (1 mM) (Sigma-Aldrich), Ellman’s reagent (0.05 mM) and 2 mM MgCl_2_ in 20 mM NaH_2_PO_4_ (pH 7.3) buffer, forming an active nucleoprotein filament comprising about one helical turn (11). Ellman’s reagent was added to prevent the formation of intermolecular disulphide bridges in RecA while it does not reduce the pre-formed disulphide bridge (i.e. the disulphide bridge in the sulpho**-**SBED reagent). Based on the observation that the ends of RecA* filaments associate by hydrophobic and ionic interactions (23), we predict that longer nucleoprotein filaments were formed. Sulpho-SBED derivatives of the wt and QM LexA (LexAQM) were prepared as reported (24). 300 μg of LexA in labelling buffer (0.5 mg/ml in 20 mM Na-phosphate buffer pH 7.3, 200 mM NaCl) and sulpho**-**SBED (in DMSO) were mixed in a 1:1.5 molar ratio and stirred for 30 min. Non-reacted sulpho**-**SBED was then dialysed out overnight. All manipulations were performed in the absence of light. Next day, to 300 μg each of sulpho**-**SBED**–**LexA derivative, wt and QM repressors, each at 3.9 µM final concentration, equimolar amounts of active nucleoprotein filament, consisting of one helical turn, at 26.0 µM final concentration, were added to each repressor in 20 mM Na-phosphate buffer pH 7.3, 200 mM NaCl, 1 mM ATP-γ-S and 2 mM MgCl_2_ to a final volume of 1.6 ml. Immediately, the solutions were irradiated for 5 min by five 15 W UV lamps at 312 nm from a distance of 4 cm (Supplementary Figure S1A). In the control experiment, 100-fold excess of unlabelled wt LexA over sulpho**-**SBED**–**LexA was added.

### Analysis of conjugates

Sulpho-SBED**–**LexA**-**labelled samples were analysed on SDS–PAGE under reducing conditions, and gels were Western-blotted in a tank for 90 min at 200 mA to PVDF membranes in Towbin buffer (25 mM Tris/HCl, 192 mM glycine, 0.1% (w/v) SDS and 20% (v/v) MeOH). On the blots, the biotinylated adducts were detected using streptavidin–HRP and BM Chemiluminescence Western blotting detection system according to the manufacturer’s instructions (Supplementary Figure S1B).

### Isolation of the biotinylated peptides

The sulpho**-**SBED**–**LexA**–**RecA* complexes were cleaved at 37°C with two sequential 1-μl additions of 1% (w/w) α-chymotrypsin at 30-min interval. To isolate the biotinylated peptides, the digested mix was applied to an avidin-affinity column. The column was then washed exhaustively with the labelling buffer, followed by milliQ water. The retained biotinylated peptides were eluted with 0.1% (v/v) trifluoroacetic acid (TFA) in milliQ water (solution A). Peptides were then separated by reversed-phase high-pressure liquid chromatography (RP-HPLC) using Agilent C18 column (150 × 4.6 mm) equilibrated with 0.1% (v/v) TFA in milliQ water (solution A). The column was washed with 5 ml of solution A, and the bound peptides eluted at a flow rate of 0.5 ml/min with a gradient of solution B (90% (v/v) acetonitrile and 0.1% (v/v) TFA in milliQ water) in solution A, from 0% to 100% of B in 40 min. Absorbance was monitored at 215 nm. Separated biotinylated peptides were vacuum dried and submitted to cycles of automated Edman degradation (Applied Biosystems Procise 492 A sequencing system cycles). Only sequences of cross-linked LexA–RecA* peptides were taken into consideration for modelling the complex (Supplementary Figure S1C).

### Modelling procedures

The atomic co-ordinates of RecA*, comprising six RecA protomers (pdb ID: 3CMU) (8), and of LexA, with the first subunit well-ordered throughout and the second disordered in NTD (pdb ID: 1JHH) (7), were obtained from the Protein Data Bank. Full-length wt LexA, LexAQM and seven RecA protomeric filament three-dimensional models were constructed using the homology modelling programme Modeller 9v4 (25). The atom co-ordinates obtained in each separate pdb file were re-numbered as 1 to 404 for the LexA and 1 to 2295 for the RecA*. For the docking runs, the HADDOCK approach was used (20). HADDOCK is a modelling program that uses biochemical and/or biophysical interaction data (e.g. cross-linking, mutational or bioinformatic prediction data) to drive the docking procedure. Docking was performed with most of the parameters set to default using the web server version of HADDOCK with a Guru interface using only cross-linking data to drive the modelling protocol. The following parameters were changed: distance restraints were set to 12 Å, sampling parameters for water refinement to 100, RMSD cut-off for clustering to 5 Å; the C2 symmetry segment was set for the LexA. For the wt LexA–RecA* docking trials, LexA passive residues were K160 (chain A), K175 (chain A) and K159 (chain B). N163 (chain A) and K164 (chain A) were active whereas residues T150 (chain A), E154 (chain A), T150 (chain B), E154 (chain B), C129 (chain B), K6 (chain C), K8 (chain C), K256 (chain G) and S329 (chain G) were RecA passive. E156 (chain B), K19 (chain C), S240 (chain G) and R243 (chain G) were active. For the LexAQM–RecA* docking trials, LexAQM HADDOCK passive residues were K135 (chain A), K160 (chain A and B), K106 (chain B) and K175 (chain B), and HADDOCK active 161Q (chain A). RecA HADDOCK passive residues were T150 (chain A), K19 (chain C), K216 (chain G), K256 (chain G) and K302 (chain G), and HADDOCK active, K152 (chain A), E156 (chain A) and K19 (chain C). Several docking trials were performed by changing the number of the passive and active residues and by adding the mutation data during the model validation step. Finally, the models of wt LexA–RecA* and of the LexAQM–RecA* complex with the highest HADDOCK scores that is in agreement with all experimental data was selected. To construct the LexA filament on the two turn RecA*, as observed from the electron micrograph images (13), from the final model of wt LexA–RecA* complex, the co-ordinates of one LexA monomer with less interaction sites to RecA*, were subtracted. Based on the S3 symmetry, the LexA filament was constructed using homology modelling with Modeller 9v4 (25).

### Surface plasmon resonance assay

SPR measurements of the LexA(M24C) binding to chip-immobilized *tisB* operator DNA were performed on a Biacore T100 (GE Healthcare) at 25°C. The streptavidin (SA) sensor chip (GE Healthcare) was equilibrated with SPR buffer containing 20 mM NaH_2_PO_4_ (pH 7.3), 0.14 M NaCl and 0.005% surfactant P20. Approximately 25 response units (RU) of biotinylated primer S1 (5′-cgctcgagtagtaac-TEG-Bio-3′) was immobilized on the flow cells of the SA chip. The DNA fragment with the 24 bp *tisB* operator and the 15-nucleotide overhang complementary to the SA chip-immobilized primer was prepared by annealing complementary primers TisBUpspr (5′-gttactactcgagcgattactgtttatttatacagtaaa-3′) and TisBDo, mixed in 1:1.5 (mol:mol) ratio respectively, as described above. This specific DNA fragment was passed for 30 s at 5 µl min^−^^1^ across the flow cell 2 to hybridize ∼20 RU of *tisB* operator. The interaction between the LexA(M24C) in the oxidized and reduced states of wt LexA was studied by injecting 150 nM solutions of the protein in SPR buffer at 100 µl min^−^^1^ for 60 s. Dissociation was followed for 300 s. SPR experiments were performed in triplicate; a representative experiment is shown.

SPR measurements of the interaction of the full-length or the CTD (residues from Gly75 to Ile202) of the wt LexA, non-cleavable LexA(K156A), the LexAQM mutant and the NTD fragment of LexA (residues 1–69) with chip-immobilized RecA* were performed on a Biacore X (GE Healthcare) at 25°C. RecA protein (2.1 µM) was passed across 5′-biotinylated 30-mer to create approximately 1000 response units (RU) of RecA* on the flow cell 2, as described (16). Proteins were injected across the reference flow cell 1 and immobilized RecA* at 10 µl/min for 60 s. The sensor chip with bound RecA* was regenerated with 500 mM NaCl, while the 0.05% (w/v) SDS was used to additionally regenerate the flow cell 1.

## RESULTS AND DISCUSSION

### Interaction area between LexA and RecA* in the complex

**Figure 1.**
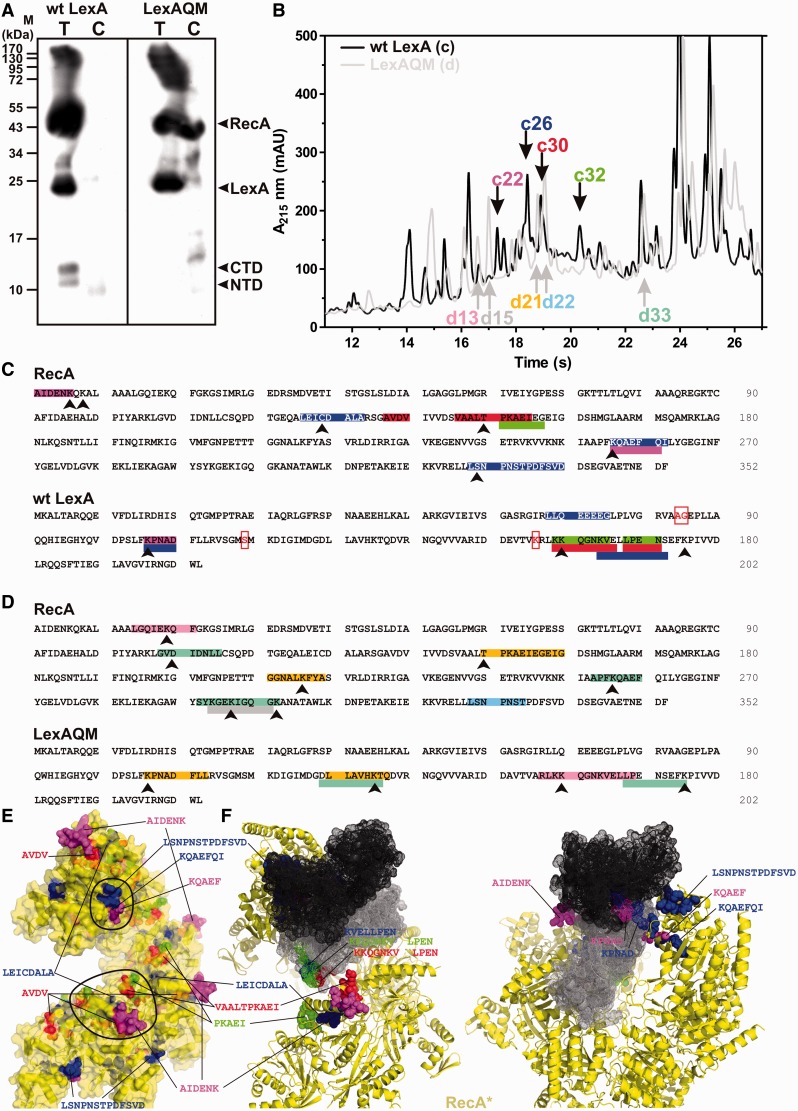
The interacting area between LexA and RecA* in the LexA–RecA* complex. (**A**) RecA* was mixed with equimolar amounts of sulpho-SBED–wt LexA or with sulpho-SBED–LexAQM in the dark, both in the absence (T) and the presence (C) of 100-fold molar access of wt LexA and immediately irradiated with UV radiation at 312 nm. Samples were analysed by SDS–PAGE under reducing conditions, transferred to PVDF membrane and the positions of biotin-containing adducts visualized by streptavidine-HRP and ECL detection, to detect label transfer to the RecA protein (Supplementary Figure S1B). (**B**) Following the α-chymotrypsin fragmentation of the photo-cross-linked complexes, wt LexA–RecA* and LexAQM–RecA*, the biotinylated peptides were enriched on avidin beads and separated by RP-HPLC. On the chromatogram the sequenced HPLC peaks are marked. (**C, D**) Sequences of the cross-linked peptides are marked on the primary structures of RecA, LexA and LexAQM. Arrows indicate the site of covalent attachment of the cross-linker. The same colour code is used as in (**B**). Red box highlights residues Ser119 and Lys 156 (the catalytic dyad) and Ala84 and Gly85 (which flank the peptide bond that is cleaved). (**E**) Segments on the RecA* structure (yellow transparent surface) cross-linked to the wt LexA are marked using the same colours as in figures (B) and (C). Identified sequences cluster in two areas from which distance restraints (encircled) were obtained to drive the HADDOCK modelling. (**F**) Two views of the wt LexA–RecA* best HADDOCK model of the complex. The identified sequences of the cross-linking LexA and RecA sites are marked by the same colours as in **B** and **C**. The LexA monomers are shown in black and grey.

In an alternative approach to mutational analysis and electron microscope studies, we mapped the interaction site between the proteins by chemical cross-linking (26). Covalently linked complexes were prepared using a photo-reactive sulpho-SBED derivative of the wt LexA, or of the non-cleavable quadruple mutant LexA(L89P/Q92W/E152A/K156A) (LexAQM) that adopts the state required for self-cleavage (7), and the RecA* nucleoprotein filament comprising one DNA helical turn (11) (Figure 1A, Supplementary Figure S1A). Repressor–RecA* conjugates were cleaved with α-chymotrypsin, the cross-linked peptides purified and identified by Edman sequencing (Figure 1B, Supplementary Figure S1C, Supplementary Table S2). The commercially available α-chymotrypsin contains traces of trypsin and since three sequential additions of protease were used, also tryptic cleavage occurred. The HPLC isolated peptides were relatively short; therefore, BioBrene was used to retain the peptide during the Edman sequencing. However, for some peptides the identities for the first two or even up to the first four amino acid residues were ambiguous. Therefore, only the unambiguously recognized amino acid residues are shown in the Figure 1C and D. Most LexA or LexAQM peptides cross-linked to the RecA* originated from the repressor’s CTD (Figure 1C–E). Furthermore, approximately half of LexA- or LexAQM-linked RecA peptides’ sequences overlap and all coincide in the three-dimensional structure of RecA*. Because the identified peptides can derive from any of the RecA* or LexA protomer surface, only the mathematically clustered combinations of surface-exposed residues were considered (Figure 1E). In this way three possible combinations of cross-linked peptides were obtained for RecA* and two possible combinations of cross-linked peptides for the LexA. Taking symmetry into consideration, only two combinations of cross-linked peptides from the RecA* were conceivable (Figure 1E and F).

### Structural insight into the key step of the bacterial SOS response

**Figure 2.**
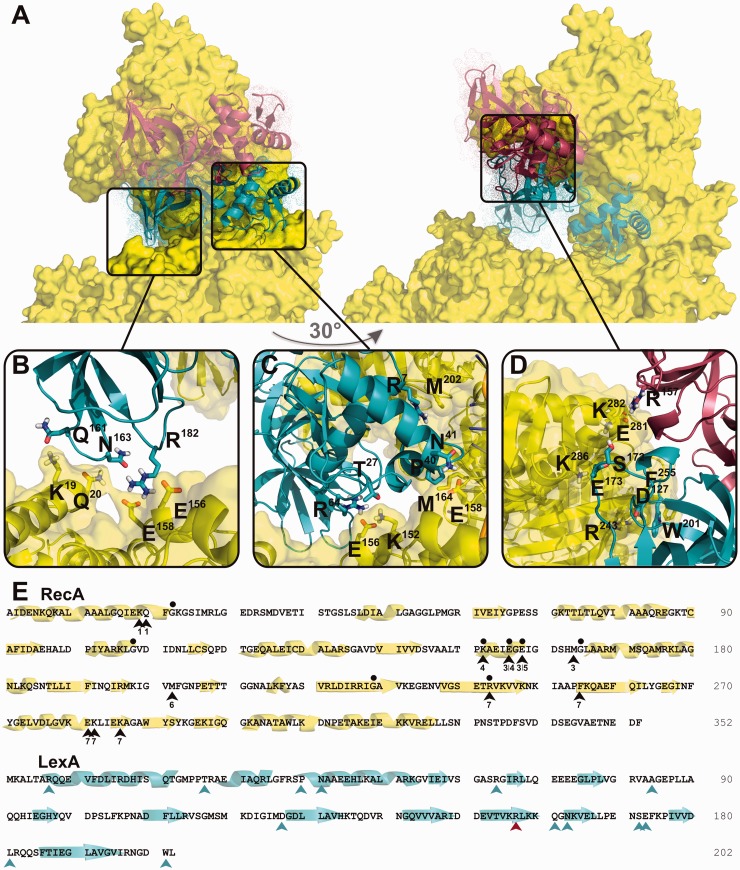
A detailed view of the LexA–RecA* interaction areas. (**A**) Two views of the complex rotated by 30° around the vertical axis (cartoon representation, one LexA monomer in cyan, the other in red) binding to seven successive RecA monomers (yellow, surface presentation). (**B–D**) Close-up view of the LexA–RecA* interaction surface. Side chains of the interacting residues are depicted. (**E**) In the RecA sequence, black arrowheads indicate the LexA-interacting residues. Number below an arrowhead indicates the RecA monomer in the RecA heptamer, which is interacting with the repressor. Black points mark those RecA residues, which mutations affected the RecA* binding to LexA but did not abolish the RecA* homologous recombination activity at residues G22, G108, K152, E156 and E158 (15,28,29) and positions of the strong contact around residues E156, G165, G229 and R243 identified by EM (13). Cyan and red arrowheads indicate amino acid residues in the respective subunit of the LexA dimer that interact with RecA*. Elements of secondary structure are designated below the RecA and LexA primary structures.

**Figure 3.**
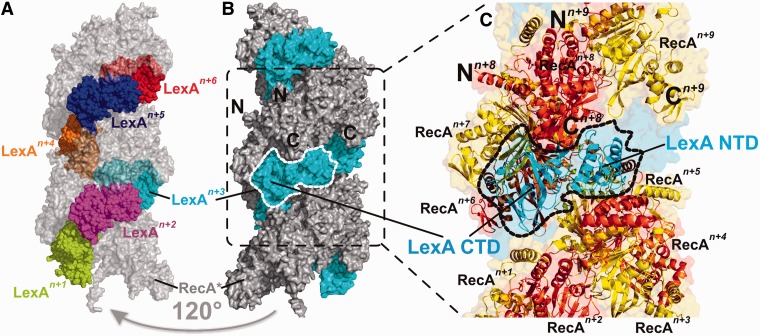
Model of the LexA–RecA* three-dimensional structure. (**A**) Six intact LexA monomers (spherical representation, each in a different colour) are docked on two turns of the RecA* (shown as grey transparent surface). (**B**) LexA–RecA* complex rotated by 120° around vertical axis relative to the view in (A). LexA monomers are presented in cyan and RecA in grey. The N- and C-termini of the two RecA monomers are marked. One of the LexA monomers is encircled by a broken line. (**C**) Detailed view of the LexA–RecA* complex. The same LexA monomer as in (**B)** is encircled. LexA CTD and NTD are indicated. Nine successive RecA monomers (presented in yellow and orange) surround one monomer of LexA. Seven RecA protomers out of nine constitute the LexA-interaction interface. The N- and C-terminai of the eighth and nineth RecA monomer are indicated.

**Figure 4.**
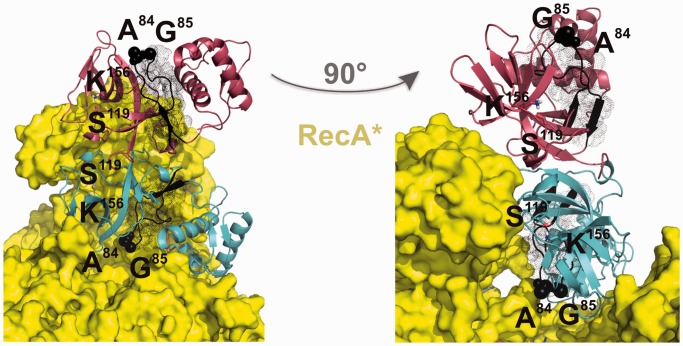
Position of the catalytic core of repressor in the LexA–RecA* complex. One monomer of LexA is coloured in cyan, the other in red. The active site Ser119/Lys156 dyad is marked as sticks, the cleavage site Ala84/Gly85 as black spheres. The cleavage site region, residues Gly75-Tyr98, is denoted as black shadow space-fill. RecA* is presented in yellow.

Using the HADDOCK approach (20), we docked the RecA* filament and wt LexA repressor structures (7,8), taking the LexA–RecA* interaction site mapping data as constraints (see Materials and Methods). The six energetically most favourable three-dimensional models of the LexA–RecA* complex were generated (Supplementary Figure S2), exhibiting an average root mean squared deviation (RMSD) of 1.4 Å for the pairs of LexA in the complexes with respect to RecA* in the complexes. Only one mode of LexA binding to RecA* (Figure 2) permitted interaction of the repressor all along the extended nucleoprotein filament (Figure 3), as inferred also from the reconstruction of LexA–RecA* electron micrographs (13). One subunit of the LexA dimer forms multiple contacts along seven successive RecA protomers, while the second subunit interacts only with the edge of the nucleoprotein filament’s deep helical groove. Hence, the cleavage site region of one repressor molecule contacts the RecA* core region while the active site of the second molecule is solvent exposed (Figure 4). Our model is thus consistent with the observation that sequential dockings of LexA dimer with RecA* are required for inactivation of both repressor subunits (16,22). *In vitro* studies demonstrated that the formation of LexA dimer and its dissociation to monomer is a slow process but when LexA is inactivated by self-cleavage, the CTD cleavage fragments dissociate faster from the LexA–LexA/CTD heterodimer (11,27). Hence, the intact subunit of the cleaved repressor heterodimer, that is, an inactive intermediate (6), could be an intracellular source of monomers that dimerize into functional repressor to accelerate reversing the repression of the SOS response following DNA damage repair.

To determine the mode of binding of the repressor to the core of RecA* when the former is in a state favouring cleavage, we generated a model of the LexAQM–RecA* complex. This model indicates that wt LexA and LexAQM converge to similar complexes with a Cα-RMSD of 4.5 Å with respect to RecA* (Supplementary Figure S3). These results lead to the conclusion that only one subunit of the repressor wedges itself into the core region of RecA*, enabling catalysis of the specific proteolytic cleavage. As RecA* stimulates inactivation of LexA in numerous species of different bacterial phyla (6), the residues composing the interaction interface may have coevolved and may differ from the here presented *E. coli* interaction sites.

### Structure-activity analysis of the LexA–RecA* complex

The proposed LexA–RecA* interaction interface is in accordance with RecA mutational studies (Figure 2E) and supports the earlier observation that multiple residues interact at the complex interface (12,14,15,28,29). Hence, loss of a single contact results in only a small defect, which can explain why mutations that specifically impair RecA-mediated cleavage of repressor have not been identified in LexA (11,12). In the LexA CTD, we modified nine amino acid residues based on the generated LexA–RecA* structure (Figure 2) or based on other energetically favourable models (Supplementary Figure S2), we modified four or five amino acid residues (Supplementary Table S1) and tested these repressor variants for self-cleavage activity (Supplementary Figure S4). Far–UV CD spectra of LexA, LexA4 and LexA5 molecules revealed comparable secondary and tertiary structures, but the spectra of LexA9 variant indicated unfolded state of the protein (Supplementary Figure S4A). Thus, we omitted LexA9 from further experiments. Under physiological conditions cleavage requires RecA*, but, *in vitro*, it can proceed rapidly, independently of co-protease at alkaline pH (30). We observed that mutations in LexA4 and LexA5 blocked or slowed down alkaline pH-induced self-cleavage but did not affect RecA*-mediated cleavage (Supplementary Figure S4B and S4C). Collectively, results indicate that it is not possible to generate a repressor variant that would exhibit substantially decreased RecA*-mediated cleavage activity and, at the same time, preserve the same self-cleavage activity.

### Operator DNA sterically blocks the binding of LexA into the RecA* core region

**Figure 5.**
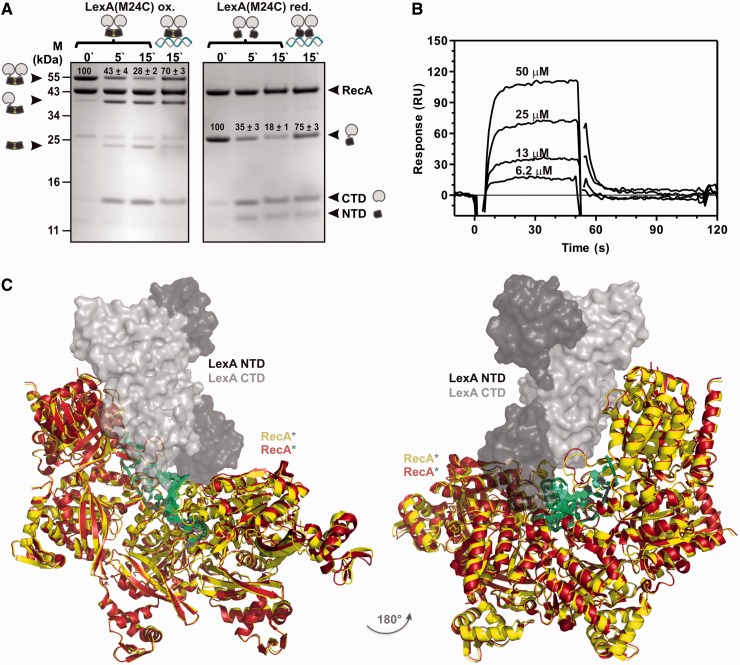
LexA DNA binding domain interacts with RecA*. (**A**) LexA in a DNA-binding conformation is inactivated on interaction with RecA*. Time course (min) of RecA*-induced proteolysis of LexA(M24C) in the oxidized, DNA-trapped state, or in the reduced, flexible state. LexA dimer, LexA monomer cross-linked to the N-terminal fragment and cross-linked N-terminal fragments are presented next to the molecular mass marker lane (M). States of the LexA(M24C), the oxidized repressor variant trapped in the DNA-binding conformation (linked NTDs) or the reduced mutant repressor variant interacting with non-specific (marked as LexA without DNA) or the specific DNA (LexA bound to DNA) are presented above the gel. The full length RecA and LexA(M24C) protomers and the cleavage forms of LexA(M24C) (CTD, NTD) are also marked. Quantification of oxidized LexA(M24C) dimers or of reduced LexA(M24C) monomers is presented on the gel above the respective band as the ratio (%) of the protein density value of the initial sample (0 min) relative to the density value obtained from the proteins after 5 or 15 min after addition of RecA*, shown with standard deviation. (**B**) SPR sensorgram of the binding of the LexA(1–69) NTD with immobilized RecA*. LexA(1–69) in concentration range from 6.2 to 60 µM was injected across the chip-immobilized ∼1000 RU of RecA* for 60 s at 10 µl/min. (**C**) Secondary DNA of the postsynaptic nucleoprotein filament, enabling exchange of strands between two homologous DNA molecules in recombination, sterically precludes interaction of RecA* with LexA. Postsynaptic RecA* (PDB ID: 3CMX) shown as RecA* in red and dsDNA in green was superimposed by RecA to the LexA–RecA* structure. The RecA* is presented in yellow and LexA in grey (CTDs) and black (NTDs).

The DNA-binding NTD of LexA can take up a variety of conformations, one of which is captured on binding the operator DNA (16). It was suggested that due to the rigidity, the DNA-bound LexA cannot interact with RecA*. Hence, the SOS response is instigated only after the dissociation of the repressor from its DNA targets (16). The LexA–RecA* structural model predicts that, in addition to the catalytic CTD, the DNA-binding NTD of the repressor’s subunit also binds the RecA* core region (Figure 2C). The LexA operator DNA could not be docked into the proposed structure of the LexA–RecA* complex, so our model suggests that operator DNA sterically precludes operator-bound LexA–RecA* complex formation. To test experimentally whether this is really so, a single cysteine LexA mutant, LexA(M24C), was engineered (Supplementary Figure S5). On oxidation, an intermolecular disulphide bond is formed, locking the LexA(M24C) dimer in the DNA-binding state (Supplementary Figure S6A) that, as determined by SPR analysis, showed similar binding to specific DNA as the wt repressor (Supplementary Figure S6B). We tested the RecA*-mediated inactivation of the oxidized LexA(M24C). The results of the RecA*-mediated LexA(M24C) self-cleavage experiment strongly indicated that the operator DNA did not inhibit the LexA–RecA* complex formation as an allosteric LexA effector but rather sterically blocked the interaction of the operator DNA-bound repressor with RecA* (Figure 5A). This supports our model, as the operator DNA cannot be accommodated into the structure of the LexA–RecA* complex. RecA* can induce self-cleavage of the repressor variant with NTDs locked in a roughly 180° orientation with respect to the oxidized LexA(M24C) (16), implying that RecA* can accommodate a repressor harbouring diverse conformations of the DNA binding domain.

To confirm that DNA-binding LexA NTD interacts with RecA* in the LexA–RecA* complex, we prepared His-tagged NTD (LexA residues from Met1 to Ile69), LexA(1–69) (7), and performed the SPR analysis. LexA(1–69) interacted with chip-immobilized RecA* in a concentration-dependent manner (Figure 5B). Furthermore, we assayed binding of the full-length or CTDs (residues from Gly75 to Ile202) of the wt LexA, non-cleavable LexA(K156A) or the LexAQM with immobilized RecA* (Supplementary Figure S7). Data exhibit that full-length repressor is needed for optimal binding to RecA*. Interestingly, CTD of the LexA(K156A) was the only protein exhibiting stable binding to the nucleoprotein filament, phenomena observed also earlier (11). Besides stimulating inactivation of LexA, the helical filament groove of RecA* serves as the site for secondary DNA binding during the homologous recombination (31). RecA* superimposed structures of postsynaptic nucleoprotein filament resolved by the X-ray crystallography (8) and of our model of the complex show that LexA repressor and secondary DNA overlap (Figure 5C). Hence, LexA–RecA* structure is consistent with the observation that these processes are competitive (31). The results support the model in which a subunit of the repressor dimer wedges into the RecA* core region.

## CONCLUSIONS

Experimental results are presented that provide original structural insights into the key step of induction of the bacterial SOS response that accelerates adaptation to antibiotics (3,4). In contrast to the recently proposed LexA–RecA* model (15), our results show that one monomer of the repressor dimer wedges into the core of RecA* and that the second monomer spans the edge of the helical filament (Figures 2 and 4). Hence, separate docking events are probably required for inactivation of both LexA dimer subunits. We propose that intact monomers of the LexA–LexA/CTD heterodimers, which we also observe in the RecA*-mediated LexA(M24C) self-cleavage experiment (Figure 5A), could be recycled into functional repressor. This adds to the understanding of the fine tuning and resetting of the SOS regulatory system following repair of DNA damage. Self-cleavage of LexA commences only after it has dissociated from its operator (16) and we show, supported by our structural model, that operator DNA sterically blocks formation of the complex between the key SOS response proteins. Most importantly, our model can be used immediately as a platform on which to design drugs to prevent accelerated spread of antibiotic resistance. It will also help in designing experiments to gain a high resolution crystal structure of LexA–RecA*.

## SUPPLEMENTARY DATA

Supplementary Data are available at NAR Online.

## FUNDING

Slovenian Research Agency [P1–0207, Z1–4071, Z1–2142, J4–2111]. Funding for open access charge: Slovenian Research Agency [P10207, P1-0198].

*Conflict of interest statement*. None declared.

## Supplementary Material

Supplementary Data
