# Supplementary material for: Structural insight into LexA–RecA* interaction
Source: Nucleic Acids Res. 2013 Aug 21;41(21):9901–10. doi: 10.1093/nar/gkt744 (PMC3834820; doi:10.1093/nar/gkt744)
Supplement: Supplementary Data [file supp_41_21_9901__index.html]

Structural insight into LexA–RecA\* interaction — Structural insight into LexA–RecA\* interaction — Supplementary Data 

# Structural insight into LexA–RecA\* interaction

## Supplementary Data

files

**Files in this Data Supplement:**

- Supplementary Data - pdf file
