# Supplementary material for: Structural insight into LexA–RecA* interaction
Source: Nucleic Acids Res. 2013 Aug 21;41(21):9901–10. doi: 10.1093/nar/gkt744 (PMC3834820; doi:10.1093/nar/gkt744)
Supplement: Supplementary Data [file supp_gkt744_nar-01880-h-2013-File007.pdf]

For Nucleic Acids Research NAR-01880-H-2013 25th July 2013

SUPPLEMENTARY MATERIAL

Structural insight into LexA-RecA* interaction

Lidija Kovačič, Nejc Paulič, Adrijana Leonardi, Vesna Hodnik, Gregor Anderluh, Zdravko Podlesek, Darja Žgur-Bertok, Igor Križaj, Matej Butala

Supplementary Table S1. Amino acid substitutions in LexA repressor variants.

**LexA mutant**	**LexA4**	**LexA5**	**LexA9**
**amino acid substitutions**	Asp127Ala<br>Gln137Ala<br>Arg140Glu<br>Phe174Ala	Gln137Ala<br>Arg140Ala<br>Lys164Glu<br>Lys175Ala<br>Arg182Glu	Asp138Ala<br>Arg140Ala<br>Arg157Ala<br>Gln161Ala<br>Asn163Ala<br>Lys164Glu<br>Glu173Ala<br>Lys175Ala<br>Arg182Glu

Supplementary Table S2. LexA-RecA* cross-linked peptide sequences (see Figure 1).

Fraction number	Cross-linked peptide sequence	sulfo-SBED reacting residues
c22	KQAEFQ (RecA)<br>KPNAD (LexA)	RecA K256<br>LexA K106
	AIDENK (RecA)	RecA 6
c26	LEICDALAR (RecA)<br>KVELLPEN (LexA)	RecA C129<br>LexA K160
	KQAEFQI (RecA)<br>KPNAD (LexA)	RecA K256<br>LexA K106
	LSNPNSTPDFSVD (RecA)<br>LLQEEEEG (LexA)	RecA K329
c30	AVDV (RecA)<br>VAALTPKAEI (RecA)<br>KKQGNKV--LPEN(LexA)	RecA T150<br>LexA K160
c32	PKAEIEG (RecA)<br>KKQGNKV--LPEN (LexA)	RecA T150<br>LexA K160
d13	LGQIEKQF (RecA)<br>RLKKQGNKVELLP (LexA)	RecA K19<br>LexA K160
d15	KGEKIGQG (RecA)	RecA K297
d21	TPKAEIEGEIG (RecA)<br>LLAVHKTQ (LexA)	RecA T150<br>LexA K135
	GGNALKFYA (RecA)<br>KPNADFLL (LexA)	RecA K216<br>LexA K106
d22	LSNPNST (RecA)	ambiguous
d22	GVDIDNLL (RecA)<br>DLLAVHKT (LexA)	RecA D110<br>LexA K135
	APFKQAEF (RecA)<br>LPENSEFK (LexA)	RecA K256<br>LexA K106
	SYKGEKIGQGK (RecA)<br>LPENSEFK (LexA)	RecA K297, K302<br>LexA K106

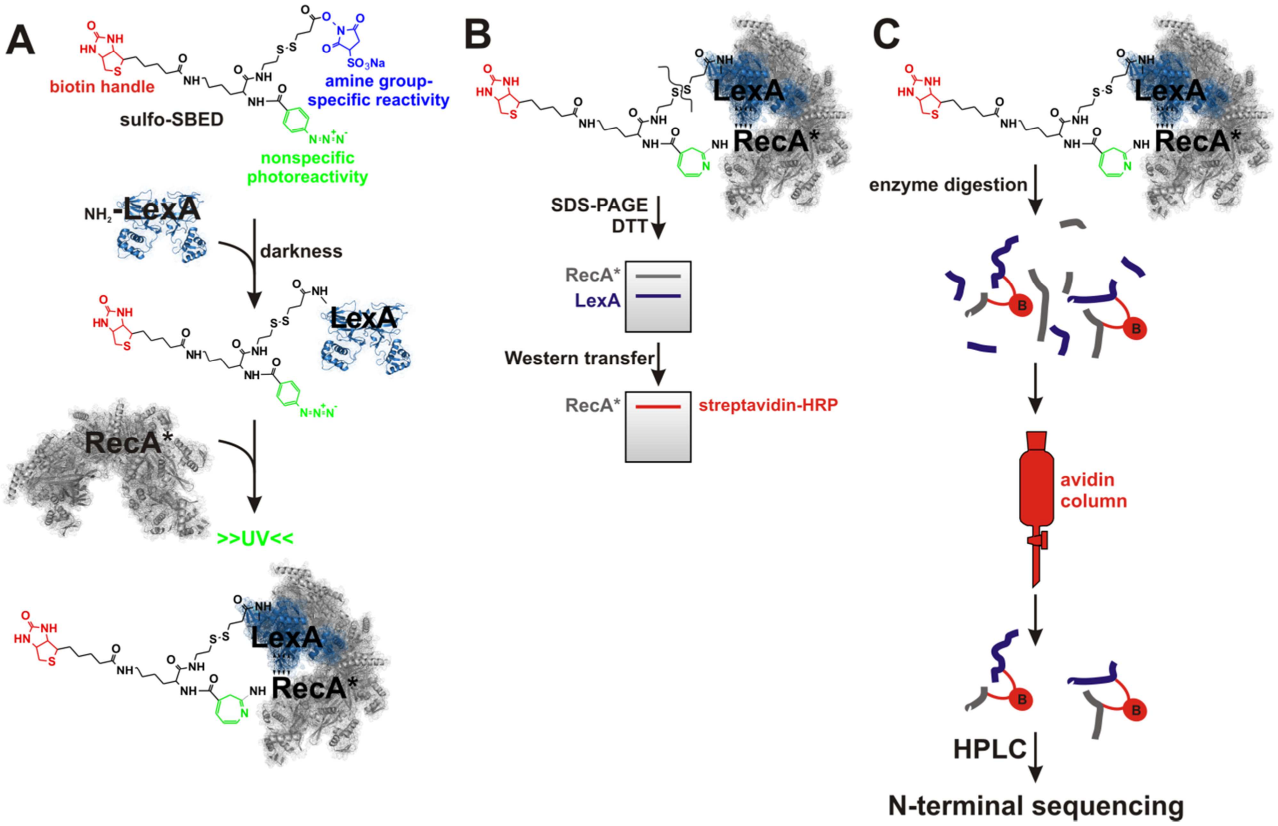

Supplementary Figure S1. Schematic representation of (A) the preparation of sulfo-SBED-LexA-RecA* conjugate, (B) analysis of the conjugate and (C) isolation of the biotinylated peptides.

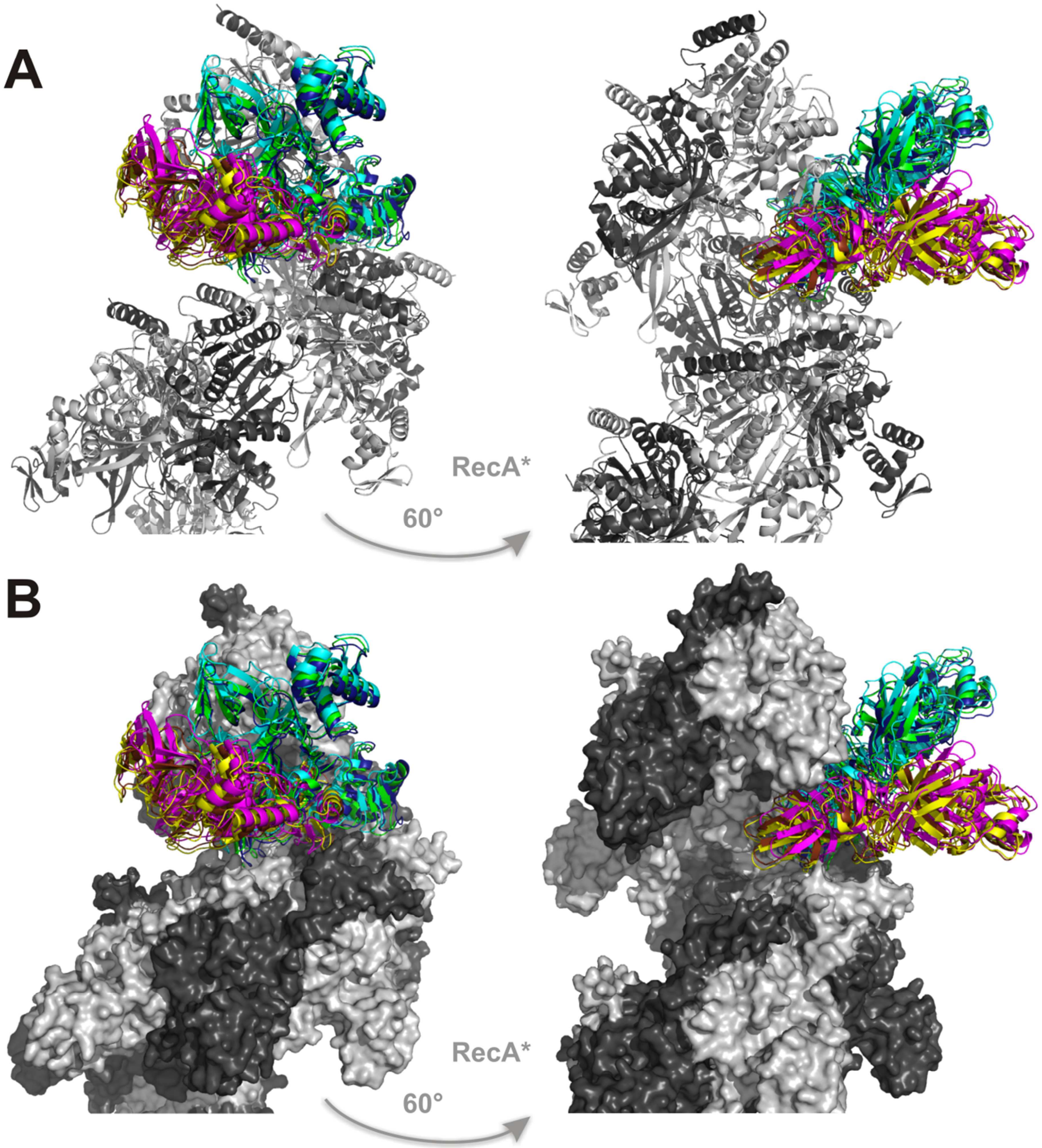

Supplementary Figure S2. The six energetically most favourable models of the LexA-RecA* obtained by the HADDOCK approach. LexA dimers are shown with different colours and RecA* is shown in cartoon (**A**) and in a surface (**B**) representation with RecA protomers in gray and black. From these models it can be seen that one subunit of LexA dimer wedges into the RecA* core region. The generated models are positioned in two clusters according to the position of the LexA repressor in complex with RecA*.

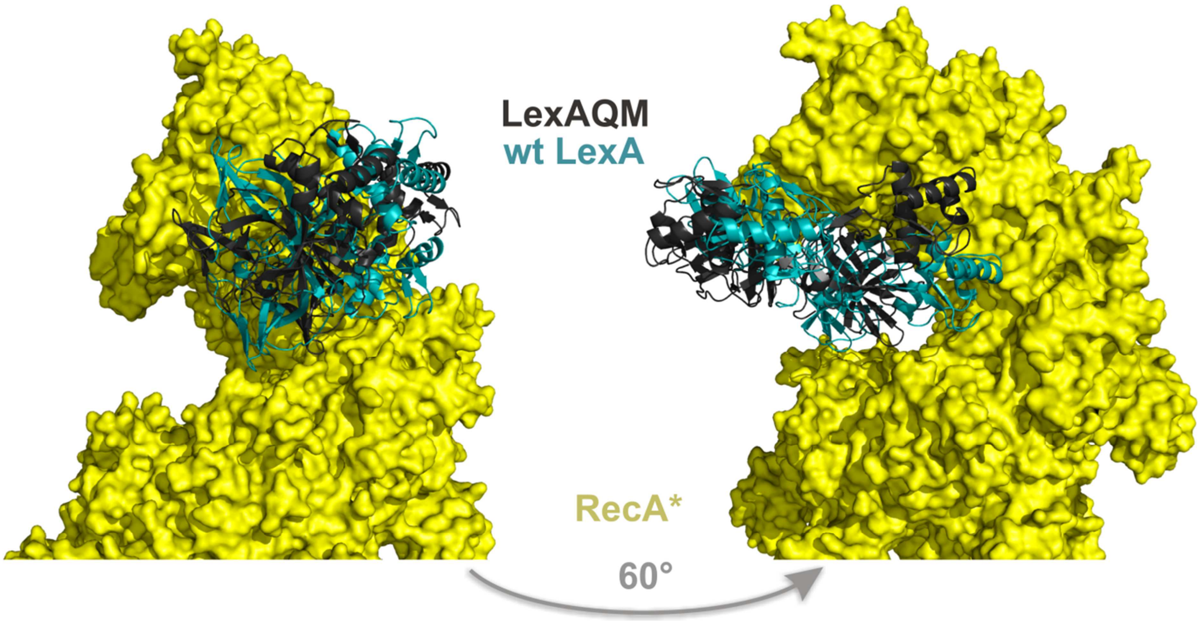

Supplementary Figure S3. Comparison of the generated LexA-RecA* and LexAQM-RecA* complexes. To obtain a structural insight into the final step of the RecA*-mediated LexA self-cleavage, we employed the quadruple mutant LexA(L89P/Q92W/E152A/K156A), LexAQM, which locks the repressor in a self-cleavage conformation (7). Due to the K156A mutation, however, the self-cleavage cannot occur. RecA* is shown in the surface representation and LexA in the cartoon representation. Two views (rotated by 60° around the vertical axis) of the superimposed wt LexA-RecA* and LexAQM-RecA* models are shown. HADDOCK modelling converges structures into structures that are very similar, exhibiting a Cα-RMSD of only 4.5 Å. One subunit of either wt LexA or LexAQM wedges into a deep helical groove of RecA*. According to the model, the repressor molecule can move slightly in the groove of the nucleoprotein filament before finally being placed in a favourable position for self-cleavage to occur.

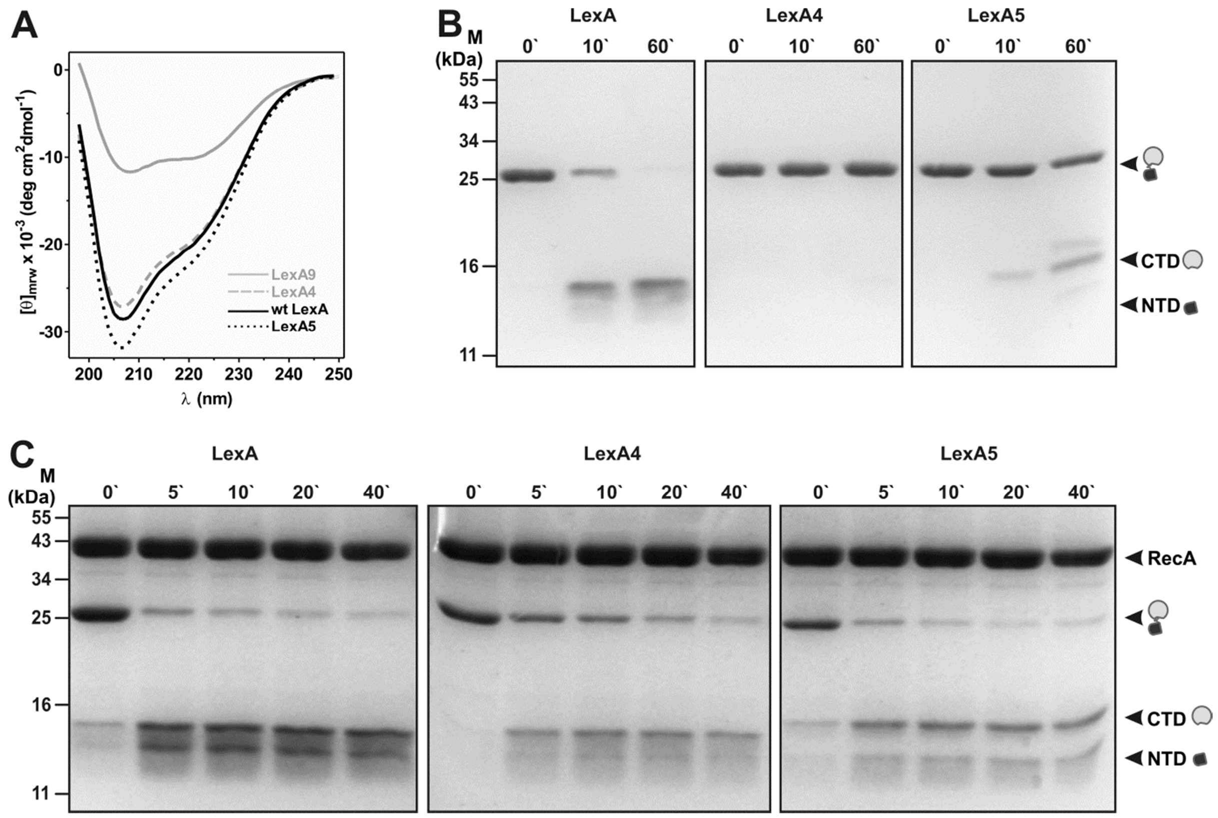

Supplementary Figure S4. Properties of purified LexA, LexA4, LexA5 and LexA9. (**A**) Far-UV CD spectra of LexA and the repressor variants. The concentration of proteins was 8 µM in 20 mM NaH_2PO_4 (pH 7.3), 0.2 M NaCl. Spectra were measured in a 0.2 mm path-length cuvette at 20°C. (**B**) Time course of LexA, LexA4 and LexA5 protein self-cleavage induced by addition of 100 mM CAPS buffer (pH 9.5). The rate of self-cleavage at alkaline pH for the repressor variants was less than that for the wt protein. Reaction mixtures were incubated for 0, 10 or 60 minutes at 37°C. (**C**) Time course (min) of RecA*-induced LexA, LexA4 and LexA5 proteolysis. They show comparable rates of self-cleavage. The cleaved LexA fragments are marked by CTD and NTD.

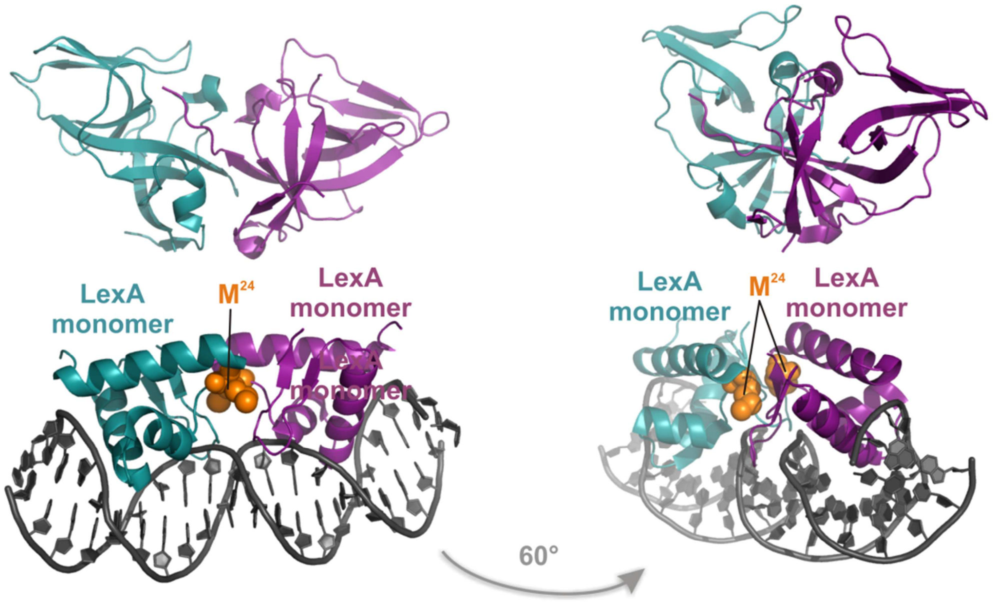

Supplementary Figure S5. Oxidation of LexA(M24C) locks the repressor dimer in a DNA-binding state. In the structure of the DNA-LexA complex (PDB ID: 3JSO), Met24 that was substituted with Cys in LexA(M24C), is shown in orange. Oxidation of Cys24 leads to formation of an intermolecular disulphide bond which locks the LexA(M24C) dimer in the operator DNA-binding state.

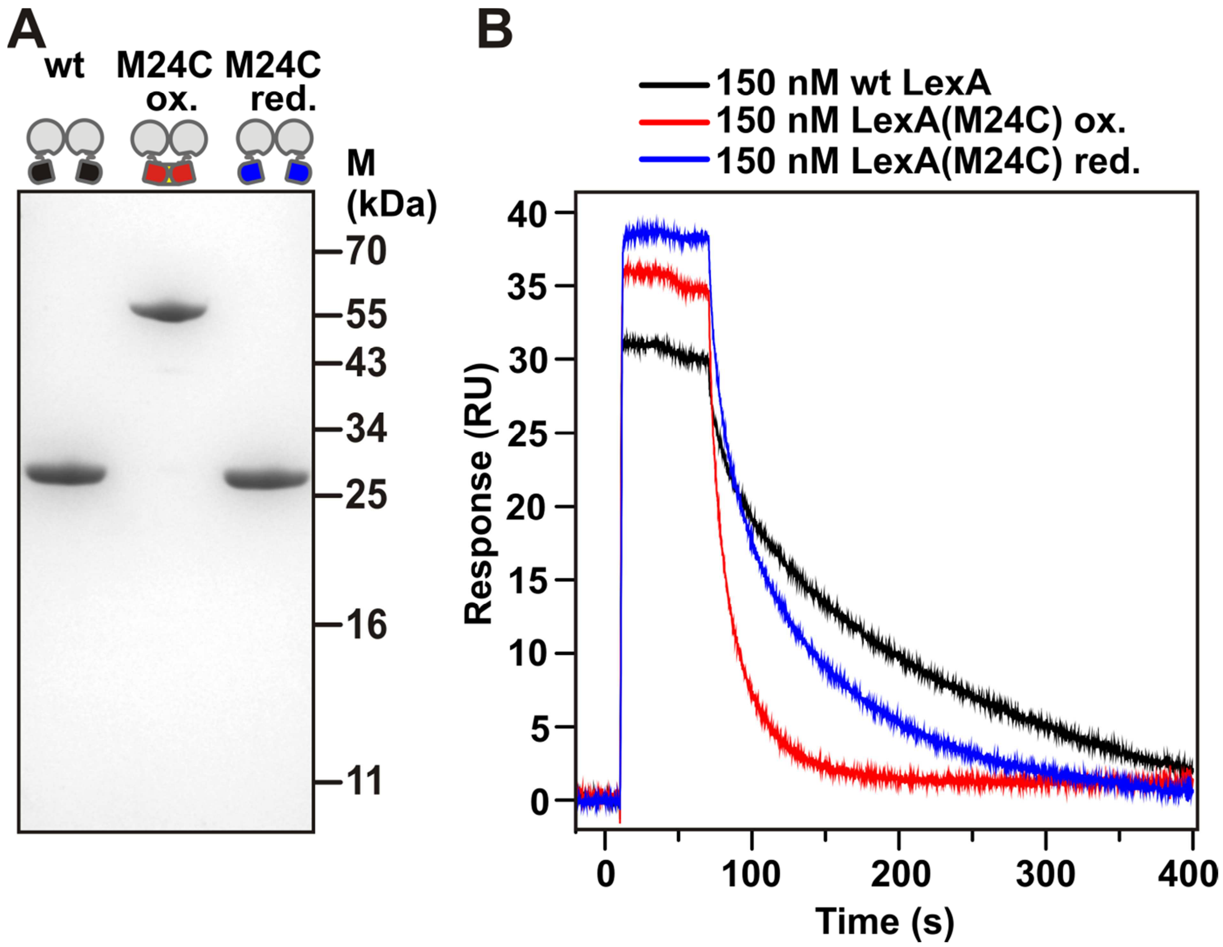

Supplementary Figure S6. *In vitro* DNA-binding properties of the wt LexA and LexA(M24C). (**A**) 12% (w/v) SDS-PAGE gel of LexA and oxidized and reduced states of LexA(M24C). The positions of molecular mass markers are denoted under M. (**B**) SPR sensorgrams of 150 nM wt LexA and LexA(M24C) interacting with the immobilized 20 response units (RU) of the *tisB* operator DNA fragment. Purified protein was injected across the chip for 60 seconds and the dissociation followed as shown.

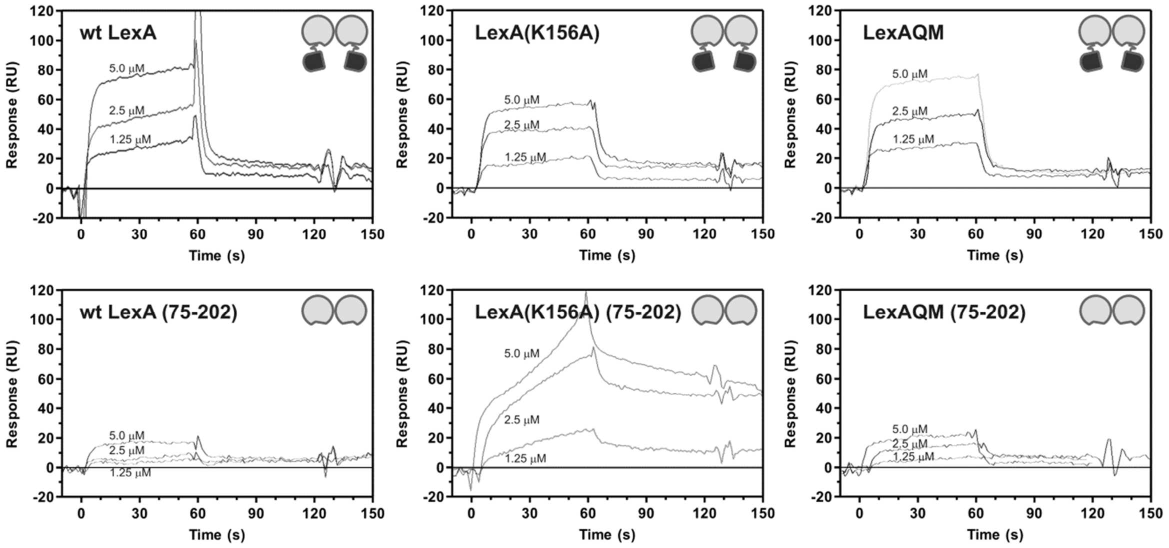

Supplementary Figure S7. Interaction with RecA* of full-length and CTD fragments (marked 75-202) of wt repressor and its variants. Repressor derivatives in a concentration range of 1.25 to 5.0 µM were injected across ~1000 RU of the chip-immobilized RecA* for 60s at 10 µl/min.
